# High Throughput Identification of Antimicrobial Peptides from Fish Gastrointestinal Microbiota

**DOI:** 10.3390/toxins9090266

**Published:** 2017-08-30

**Authors:** Bo Dong, Yunhai Yi, Lifeng Liang, Qiong Shi

**Affiliations:** 1 BGI Education Center, University of Chinese Academy of Sciences, Shenzhen 518083, China; dongbo@genomics.cn (B.D.); yiyunhai@genomics.cn (Y.Y.); lianglifeng@genomics.cn (L.L.); 2 Shenzhen Key Lab of Marine Genomics, Guangdong Provincial Key Lab of Molecular Breeding in Marine Economic Animals, BGI Academy of Marine Sciences, BGI Marine, BGI, Shenzhen 518083, China; 3 Laboratory of Aquatic Genomics, College of Life Sciences and Oceanography, Shenzhen University, Shenzhen 518060, China

**Keywords:** antimicrobial peptide (AMP), fish gastrointestinal microbiota, high throughput identification, AMP-producing bacteria

## Abstract

Antimicrobial peptides (AMPs) are a group of small peptides, which are secreted by almost all creatures in nature. They have been explored in therapeutic and agricultural aspects as they are toxic to many bacteria. A considerable amount of work has been conducted in analyzing 16S and metagenomics of the gastrointestinal (GI) microbiome of grass carp (*Ctenopharyngodon idellus*). However, these datasets are still untapped resources. In this present study, a homologous search was performed to predict AMPs from our newly generated metagenome of grass carp. We identified five AMPs with high similarities to previously reported bacterial toxins, such as lantibiotic and class II bacteriocins. In addition, we observed that the top abundant genus in the GI microbiota of the grass carp was generally consistent with the putative AMP-producing strains, which are mainly from *Lactobacillales*. Furthermore, we constructed the phylogenetic relationship of these putative AMP-producing bacteria existing in the GI of grass carp and some popular commercial probiotics (commonly used for microecologics), demonstrating that they are closely related. Thus, these strains have the potential to be developed into novel microecologics. In a word, we provide a high-throughput way to discover AMPs from fish GI microbiota, which can be developed as alternative pathogen antagonists (toxins) for microecologics or probiotic supplements.

## 1. Introduction

Antimicrobial peptides (AMPs) are a group of small peptides, which are secreted by almost all creatures in nature. They are being explored with regards to their potential therapeutic and agricultural uses as they are toxic to many bacteria. These AMPs synthesized in the ribosomes of bacteria are also called bacteriocins, which normally present antibacterial activity towards closely-related strains, although it has been reported that a broad range of antimicrobial activity occurs in some bacteriocins [1]. Those bacteriocins of lactic acid bacteria (LAB) have raised a considerable amount of attention nowadays [2]. Nisin, the first bacteriocin applied to therapeutics without any side effects, can effectively inhibit the growth of Gram-positive (G+) bacteria, including many members of *Lactobacillus*, *Pediococcus*, *Leuconostoc*, *Micrococcus*, *Staphylococcus*, *Listeria*, and *Clostridium* [3].

It is well known that the majority of G+ bacteria are beneficial to intestinal health. *Lactobacillus* is the largest genus of the LAB group (with over 50 species in total), contributing important metabolic reactions to the production of cheese, yogurt, and other dairy products [4]. They are commonly found in the oral, vaginal, and intestinal regions of many animals. These organisms have also been shown to stimulate the immune system and have antibacterial activity against intestinal pathogens, which indicates that they may be useful as probiotics. *Pediococcus acidilactici* is usually used to regulate gastrointestinal (GI) floras as they balance microecology by acid production [5]. *Bacillus subtilis* is a commonly applied microecologic in agriculture feed additives and food products [6]. *Streptococcus* contains many commensal floras located at the epidermis, respiratory tract, and gut, such as beneficial *S. thermophilus* and pathogenic *S. pyogenes* [7].These probiotics are active against microorganisms by antagonism in vivo, particularly against those closely-related competing bacteria. Some of them have a broader inhibitory spectrum in certain conditions, while some of them have a rather narrow spectrum. Additionally, their metabolites improve the activity of acidic proteases and prevent the production of harmful molecules.

The grass carp, *Ctenopharyngodon idellus*, is an herbivorous freshwater fish of the Cyprinidae family and a commercial species widely cultivated in China. Many previous works have focused on employing the 16S rRNA method in analyzing the GI microbiota of grass carp [8]. However, in order to obtain a more accurate phylogenetic composition, the whole metagenomic data should be put to use. Meanwhile, since antibiotic abuse in aquaculture has caused many problems, such as pathogen resistance and food contamination, we are trying to screen new bacterial species with a high efficacy of AMP production as potential probiotics to combat aquatic pathogens.

In this research, we sequenced the whole metagenome of the GI microbiota in grass carp and a high-throughput homologous search was performed to predict AMPs. In addition, the phylogenetic relationship of some genera in grass carp GI microbiota, putative AMP-producing strains and commonly used probiotics for microecologics were described. Interestingly, these newly identified AMP-producing strains have the potential to be developed into novel microecologics or probiotic supplements.

## 2. Results

### 2.1. Summary of Achieved Metagenome Datasets

**Figure 1:**
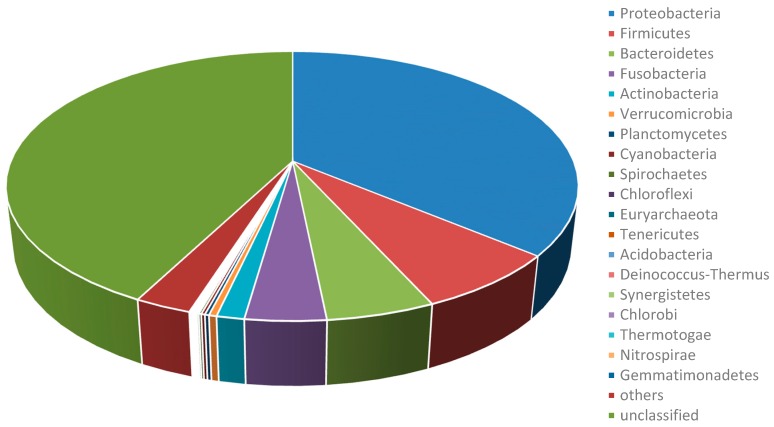
Relative abundance of phyla annotated in the metagenome of grass carp.

In total, 11 gigabase (Gb) of raw data were generated from a pool of sequencing data of eight GI samples (see more details in Section 4.1). After filtering the host contamination, 218 megabase (Mb) of metagenome data were assembled. Finally, from these acquired data, a total of 4966 species, 1453 genera, 378 families, 178 orders, 76 classes, and 54 phyla were annotated (Table S1). The GI microbiome of grass carp are dominated by members of phyla Proteobacteria (36.12%), Firmicutes (7.14%), Bacteroidetes (5.16%), Fusobacteria (3.82%), and Actinobacteria (1.31%; Figure 1). The well represented genera are *Aeromonas* (19.02%), *Shewanella* (3.79%), *Cetobacterium* (2.96%), *Bacteroides* (1.60%), and *Clostridium* (0.81%), respectively (Figure 1, Table S1).

### 2.2. AMPs Identified in the Metagenome

**Figure 2:**
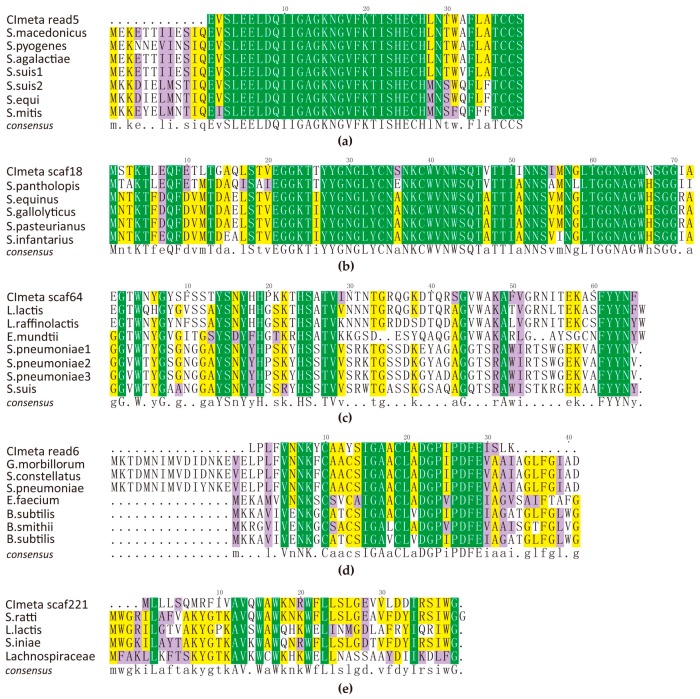
Multiple sequence alignment of identified AMPs with public bacteriocins in the NCBI. (**a**) lantibiotic; (**b**) Pediocin-like (Class IIa); (**c**) Lactococcin 972 (Class IIc); (**d**) SubtilosinA (Class IIc); (**e**) Aureocin-like (Class IId). Please note that with the exception of (**b**), the sequences seem to be incomplete, which may be due to missing reads in the assembly procedure. Similar amino acids of at least 50% were shaded in purple, 80% were shaded in yellow, and those with same characteristics were shaded in green.

Based on sequence similarity, we identified five AMPs by homologous alignment (Figure 2). However, we could not assert the exact strains for producing them, although four of them seem to be incomplete. Meanwhile, we employed BLASTP to search against public databases in NCBI and found that they are novel bacteriocins except for lantibiotic (Table 1). These putative AMP-producing bacteria, summarized in Table 1, will be useful for further screening and validation of potential probiotic supplements.

A 39 amino acid (aa) fragments identified from filtered reads of current metagenome are exactly the same as the lantibiotics of *Streptococcus* spp., including *S. macedonicus*, *S. pyogenes*, and *S. agalactiae*. This post-translationally modified peptide with characteristic thioether amino acids represents a large group of promising candidates for future applications as it can be a good template for improving lanthipeptide analogs or introducing thioether cross-links into reported therapeutics [9]. The mature peptide GKNGVFKTISHECHLNTWAFLATCCS belongs to lacticin 481-like peptides of class I of lantibiotics, which are LanM modified globular peptides with a classification into class I type B lantibiotic [10]. However, their structures require further confirmation. 

The predicted complete sequence of a pediocin-like bacteriocin belongs to class IIa as it has a highly conserved consensus sequence YYGNGX_2_CX_4_CXVX_n_ [11] within its N-terminal domain. This 49-aa mature peptide seems to be produced primarily by strains of genus *Streptococcus* [12]. Lactococcin 972 (lcn972) was first isolated from *Lactococcus lactis* subsp. *lactis* IPLA 972 [13]. Our predicted lcn972-like peptide also has a high similarity to lcn972 and thus, we speculate that it may come from strains of the genus *Lactococcus*. Furthermore, members of this family tend to be associated with transmembrane putative immunity proteins related to cellular processes, toxin production, and resistance. It has been proven that lcn972 was the first non-lantibiotic bacteriocin that specifically interacts with the cell wall precursor lipid II [14], which was categorized into subclass IIc [11]. 

The predicted subtilosin A-like bacteriocin is another class IIc member identified by us. Subtilosin A is a group of antilisterial bacteriocins previously reported only produced by *Bacillus subtilis*, before being found in many other species [15]. Aureocin-like type II bacteriocin is a small family usually encoded on a plasmid. Characteristically, the members are small, cationic, rich in Lys and Trp in addition of bringing about a generalized membrane permeabilization leading to leakage of ions. The family includes aureocin A, lacticins Q and Z, BhtB, as well as an archaeal member [16,17]. However, our predicted BhtB-like seems to be very special compared with others. 

### 2.3. Phylogenetic Analysis

**Figure 3:**
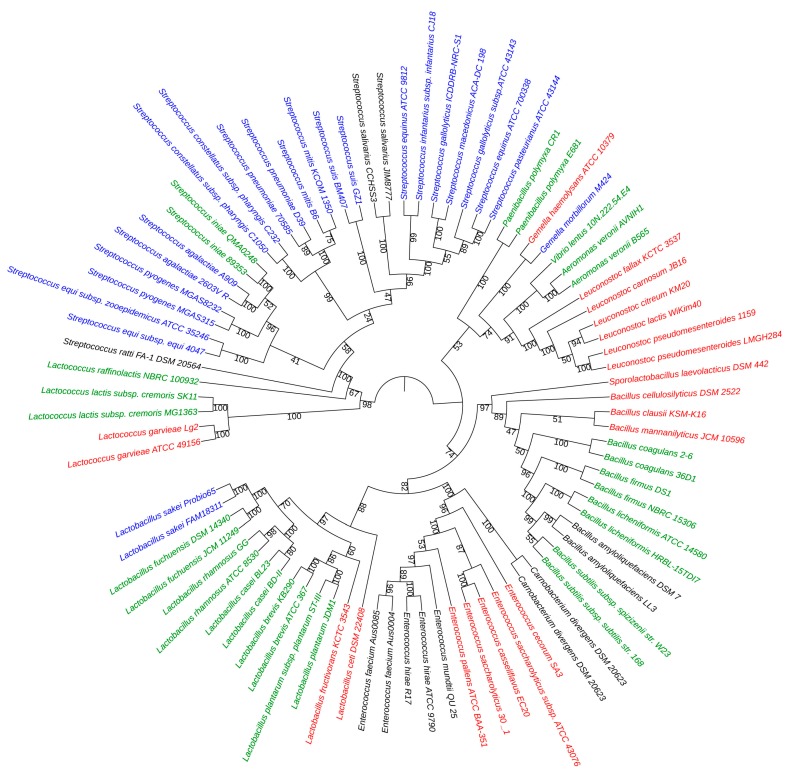
Phylogenetic tree of AMP-producing microbiota. Commercial probiotics are green, top abundant bacteria of class *Bacilli* in grass carp GI are red, and the putative strains producing our predicted AMPs are blue.

We could not determine the unique strains for these predicted AMP sequences. However, in order to evaluate the utility for potential probiotics in grass carp according to close relations between known probiotics and these putative AMP-producing bacteria, we constructed a phylogenetic tree (Figure 3) using microbiota from annotated top abundant species in class *Bacilli*, top bacterial hits in the NCBI by searching from our predicted AMP sequences, and those commonly-used commercial probiotics in microecologics.

Three *Lactococcus* spp. exist in the GI of grass carp, with both *L. raffinolactis* and *L. lactis* being putative AMP-producing probiotics. However, several *Lactococcus* strains are reported as pathogens. Thus, only some strains have the potential to become probiotics in aquaculture as agonists of *S. aureus*, while *L. garvieae* SYP-B-301 has been employed as a feed additive [18,19,20,21]. In *Streptococcus* spp., *S. iniae* I1 was selected as predominant LAB in the intestines of young common carp, which is consistent with what we observed. Streptococcaceae was the most plentiful family under the order *Lactobacillales*, followed by Lactobacillaceae and Enterococcaceae families. *Streptococcus* was the 7th most abundant genus in the identified 1453 genera, while *Bacillus* was the 11th, *Lactobacillus* was the 42nd, and *Enterococcus* was the 46th. It seems that a large amount of *Streptococcus* spp. in the GI are predicted to be AMP-producing species (Figure 3) and *S. agalactiae* with a closest relationship to *S. iniae* (a previously applied probiotics) can be a probiotic candidate. 

Meanwhile, *Gemella morbillorum* M424, a subtilosin A-producing strain closely related to some probiotics, may be beneficial to the intestine. In addition, while probiotics *Bacillus coagulans*, *B. firmus*, *B. licheniformis*, and *B. subtilis*, were found at relatively low levels, the predominant species *B. cellulosilyticus*, *B. clausii*, and *B. mannanilyticus* (Table 1) may play an important alternative role in the GI. A similar situation occurred for *Lactobacillus* spp. as *L. sakei*, *L. fructivorans*, and *L. ceti* may be helpful. *Enterococcus* spp. are another superior resource, with their functions waiting to be explored.

In summary, based on our phylogenetic tree (Figure 3), we speculate that these bacteria closely related to some known probiotics are promising candidates for novel AMPs or applicable probiotics in the grass carp, such as *L. garvieae*, *S. agalactiae*, *G. morbillorum*, and *L. sakei*. Furthermore, these predominant species of *Lactobacillus* and *Bacillus* are also valuable genetic resources for further investigation.

## 3. Discussion

In commercial aquaculture, the stressful conditions fish are exposed to usually result in a decrease in production and increase the risk of infectious diseases. However, resistance from antibiotic abuse has encouraged the scientific community to search for alternatives to antibiotics. Our present strategy to identify the potential AMPs in fish GI microbiota is an alternative way to overcome the deficiency of antibiotics. 

In this study, we identified five AMPs (pediocin, lactococcin 972, subtilosin A, and aureocin-like bacteriocins) in the GI of grass carp. However, it is still necessary to confirm the exact strains producing these AMPs. Our analysis suggested that these bacteriocins were encoded by rare members of the GI microbiota or those that have not been previously identified as important bacteria. Furthermore, we predicted the microbiota that may produce our five identified AMPs from the GI metagenomics of *C. idellus* and constructed an interesting phylogenetic tree for them. It revealed that many commensal organisms in grass carp, including three species of *Lactococcus* (*L. raffinolactis*, *L. lactis*, and *L. garvieae*) as well as many members of *Streptococcus*, *Bacillus*, *Lactobacillus*, and *Enterococcus*, are potential probiotics in grass carp, as they are closely related to the known strains used in microecologics and consistent with other characterized probiotics [22,23]. 

Interestingly, all the predicted bacterial species belong to the class *Bacilli*. In fact, antimicrobial compounds produced by *Bacilli* and isolated from GI of Japanese costal fish [24] and Indian major carp [25] have been suggested as bio-control agents, while antimicrobial peptides or bacteriocins have received attention as an alternative tool to control colonization of pathogenic bacteria in fish intestine [26]. It can be inferred that these *Bacilli* are dominant strains in many animals and other metabolites certainly have irreplaceable functions in GI. Actually, apart from G+ bacteria derived bacteriocins, antimicrobial metabolites from G− bacteria have a rather high concentration in antimicrobial providers to protect their host. In *Pseudoalteromonas*, bacteriocins are almost large proteins (>100 kDa) and their strains are of great ecological significance as part of the resident microbiota or antimicrobial metabolite producers [27]. That may explain why class γ-Proteobacteria is the largest group in the GI of grass carp (Table S1). 

GI bacteria can be variably changed by components of food or cultural media, interaction with enterocytes, water temperatures, or seasons [28,29,30]. A healthy gut is always kept in a dynamic equilibrium, which requires the coordination of many factors [29]. It seems that each bacterial species has its own natural enemies in GI and it restricts other competitors in a reasonable condition by secreting toxins such as AMPs. Their abundance in GI flora is surely in accord with their functions in this complex bacteria community. Once their balance is disrupted, diseases will occur in the host. Antibiotics are commonly abused in animal agriculture, thus causing the emergence of antibiotic-resistant bacteria. An alternative way for applying bacteriocins is to deliver some AMP-producing microbiota directly through prophylactic administration of probiotics, as long as they can stably survive in the intestine and maintain physiological homeostasis.

The rapid advancement of next-generation sequencing (NGS) has accelerated our pace of identifying novel probiotics in fish GI. Previously reported bacteriocins are usually purified by traditional isolation [31]. There are more and more approaches being developed to dig out more bacteriocins in nature, including Bacteriocin Operon and gene block Associator (BOA; [32]), Hidden Markov Model (HMM) [33], and BAGEL2 [34]. These genome mining bioinformatic tools facilitate our work in discovering more useful functional genes by fully utilizing the big data in the post-genomics era [35]. They have a great potential to look into the indicators of many diseases or the prediction of novel AMPs in metagenomes of animals. Thus, this improves the digestive conditions of fish and feed efficiency. This also provides opportunities to better determine the specific types of bacteriocins and bacterial species can be found in different environmental niches through the investigation of metagenomic data. Exploiting antimicrobial-producing resources to shape the host-associated microbiota along with high-throughput sequencing may elucidate the roles of different strains for host defense.

This is the first big-data-based study looking for AMPs and AMP-producing bacteria in the GI metagenome of grass carp. There is a great temptation to connect related microbiota that may produce bacteriocins and abundant bacteria groups with commonly acknowledged probiotics. Future studies should focus on AMPs in fish GI and bacterial strains producing them, thus offering efficient probiotic supplements in agriculture and feed industry. Their basic characteristics are worthy of further investigation and their physiological roles in interacting microbiota remain to be elucidated. Finally, in vivo experiments are required to determine the ecological significance of the association between predicted potential probiotics and their host macro-organisms. The major issue is to determine whether AMP-producing microbiota provide a real benefit to their host, especially in the context of pathogenic events. In such a case, they may stand out as the next generation of probiotics of economic importance in aquaculture. 

## 4. Materials and Methods

### 4.1. Sample Collection

Pond-cultured grass carps were fed twice a day with a commercial feed (crude protein ≥ 30.0%; crude fiber ≥ 12.0%; crude ash ≤ 15.0%; calcium 0.4–2.5%; phosphate ≥ 0.7%; salt 0.3–1.2%; moisture ≤ 12.5%; and lysine ≥ 1.2%) from Shenzhen Alphafeed Co. Ltd., Shenzhen, China. They were originally collected from a local hatchery in Shenzhen, Guangdong, China by trawl netting. These cultured fish, with an approximate size of 1–2 kg, were selected. The body surface of these fish was rinsed with sterile distilled water and subsequently, 70% ethanol was used to reduce contamination. The GI was dissected aseptically from their abdominal cavity, while the GI content and the epithelial GI mucosa were squeezed out for a separate harvest. The GI contents from eight individuals were obtained individually and stored at −80 °C before use.

All experiments were performed in accordance with the guidelines of the Animal Ethics Committee and were approved by the Institutional Review Board on Bioethics and Biosafety of BGI (No. FT15103) on 6 July 2016.

### 4.2. Assembly and Annotation of the Metagenome

Eight samples were separately sequenced by an Illumina Hiseq PE150 platform at BGI, Shenzhen, China. The pool of metagenomic data were assembled by MegaHIT software [36]. Analysis was implemented by Guangdong Magigene Biotechnology Co., Ltd, Guangzhou, China. Annotation of deduced amino acid sequences was performed through BLASTP against the public Nr database with E ≤ 1 × 10^−3^ by KAIJU [37]. To determine the accurate phylogenetic composition of GI microbiota, we assigned all the metagenomic reads to prokaryotic reference genomes that have been submitted to the genome database of NCBI using BLASTN with default parameters. The aligned reads with sequence similarity ≥75% were filtered by BLAST against the metagenome of grass carp.

### 4.3. Prediction and Identification of AMPs

We applied a homology search with the reported AMPs from the newly established *C. idellus* metagenome datasets. These previously validated AMPs were retrieved from online Antimicrobial Peptides Database (APD3) [38] and were used as queries. Assembled metagenome and raw reads of grass carp GI microbiota were used as a local index database. Subsequently, we applied TBLASTN with the threshold of E-value ≤10^−5^ to run the queries against the database. Following this, the hits of nucleotides were translated into peptide sequences and submitted to NCBI using BLASTP tool for further verification. However, those AMPs only exist in eukaryotes were removed.

### 4.4. Alignment and Homology of AMPs

The obtained peptide sequences and downloaded proteins from NCBI (Table 1) were aligned by ClustalW of BioEdit [39]. Amino acid residues with conservation among sequences were shaded by TexShade [40]. 

### 4.5. Construction of the Phylogenetic Tree

We downloaded the metagenomes of 84 strains and applied their 16S rRNA sequences to construct a phylogenetic tree. First of all, MUSCLE v3.8.31 [41] was employed for multiple alignment, in which all positions containing gaps and missing data were eliminated. Subsequently, an UPGMA tree was constructed using MEGA7 [42]. Bootstrapping was carried out using 100 replicates and values are indicated on the nodes of the phylogeny. The rate variation among sites was modeled with a gamma distribution (shape parameter = 1). Finally, the phylogenetic tree was shown in iTOL [43].

## Figures and Tables

**Table 1:** Summary of known bacteriocins in the NCBI with the highest ranked hits.

Category	Identified AMPs ^1^	NCBI AMPs	GenBank Accession No.	NCBI AMP-Producing Bacterial Species
Class I (Lantibiotic)	a	McdA	ABI30227.1	*Streptococcus macedonicus*
		Streptococcin A-FF22	P36501.1	*Streptococcus pyogenes*
		Nukacin	CZT39525.1	*Streptococcus agalactiae*
		Macedocin	CZA89639.1	*Streptococcus suis*
		type-A lantibiotic	EEF65507.1	*Streptococcus suis* 89/1591
		type A2 lantipeptide	WP_041790396.1	*Streptococcus equi*
		type A2 lantipeptide	WP_033685037.1	*Streptococcus mitis*
		lantibiotic nukacin	KEO43205.1	*Streptococcus salivarius*
Class IIa (Pediocin-like)	b	Hypothetical	AND78905.1	*Streptococcus pantholopis*
		piscicolin-126	EFM26697.1	*Streptococcus equinus*
		Bacteriocin	KUE92317.1	*Streptococcus gallolyticus*
		putative piscicolin-126	KXI11412.1	*Streptococcus pasteurianus*
		infantaricin E	AHW46171.1	*Streptococcus infantarius*
		MundKS	ACI25616.1	*Enterococcus mundtii*
		leucocin C	AEY55410.1	*Leuconostoc carnosum*
		SakX	AAP44569.1	*Lactobacillus sakei*
Class IIc	c	lactococcin 972	CAA05247.1	*Lactococcus lactis*
		lactococcin 972 family	WP_061775386.1	*Lactococcus raffinolactis*
		lactococcin 972 family	WP_065096983.1	*Enterococcus mundtii*
		lactococcin 972	CTL98394.1	*Streptococcus pneumoniae*
		lactococcin 972 family	SNP59245.1	*Streptococcus pneumoniae*
		lactococcin 972 family	CWJ26067.1	*Streptococcus pneumoniae*
		lactococcin 972 family	CYW17154.1	*Streptococcus suis*
	d	sboA protein	EFV34710.1	*Gemella morbillorum* M424
		subtilosin A	EGV07582.1	*Streptococcus constellatus*
		putative subtilosin A	CVX48913.1	*Streptococcus pneumoniae*
		Hypothetical	ELB10075.1	*Enterococcus faecium*
		subtilosin A	CAD23198.1	*Bacillus subtilis*
		subtilosin A	AKP46487.1	*Bacillus smithii*
		subtilosin A	WP_087992738.1	*Bacillus subtilis*
Class IId	e	Mutacin BhtB	AAZ76605.1	*Streptococcus ratti*
		lactolisterin BU	SDR48784.1	*Lactococcus lactis*
		Hypothetical	WP_081348647.1	*Streptococcus iniae*
		Aureocin-like	SFG15527.1	*Lachnospiraceae* C7

^1^ See more details in Figure 2.

## Supplementary Materials

The following is available online at www.mdpi.com/2072-6651/9/9/266/s1. Table S1: Bacterial components in the gastrointestinal samples of grass carp.
