# Supplementary material for: High Throughput Identification of Antimicrobial Peptides from Fish Gastrointestinal Microbiota
Source: Toxins (Basel). 2017 Aug 30;9(9):266. doi: 10.3390/toxins9090266 (PMC5618199; doi:10.3390/toxins9090266)
Supplement: Supplementary file 1 [file toxins-09-00266-s001.pdf]

Supplementary Materials: High Throughput Identification of Antimicrobial Peptides from Fish Gastrointestinal Microbiota

Bo Dong, Yunhai Yi, Lifeng Liang and Qiong Shi

Table S1. Bacterial components in the gastrointestinal samples of grass carp.

Phylum	%	reads
Proteobacteria	36.119514	15,377,022
Firmicutes	7.140841	3,040,043
Bacteroidetes	5.156369	2,195,201
Fusobacteria	3.822323	1,627,263
Actinobacteria	1.310418	557,879
Verrucomicrobia	0.363349	154,687
Planctomycetes	0.199222	84,814
Cyanobacteria	0.175117	74,552
Spirochaetes	0.122346	52,086
Chloroflexi	0.093095	39,633
Euryarchaeota	0.086765	36,938
Tenericutes	0.08046	34,254
Acidobacteria	0.058371	24,850
Deinococcus-Thermus	0.049677	21,149
Synergistetes	0.031222	13,292
Chlorobi	0.023717	10,097
Thermotogae	0.020856	8,879
Nitrospirae	0.013196	5,618
Gemmatimonadetes	0.012337	5,252
Chlamydiae	0.012261	5,220
Deferribacteres	0.012226	5,205
Aquificae	0.010056	4,281
Candidatus Saccharibacteria	0.010042	4,275
Ignavibacteriae	0.009809	4,176
Lentisphaerae	0.009243	3,935
Thermodesulfobacteria	0.007923	3,373
Armatimonadetes	0.007669	3,265
Chrysiogenetes	0.006943	2,956
Fibrobacteres	0.005901	2,512
Nitrospinae	0.004696	1,999
Crenarchaeota	0.004656	1,982
Calditrichaeota	0.004237	1,804
Balneolaeota	0.003794	1,615
Candidatus Cloacimonetes	0.003772	1,606
candidate division NC10	0.003669	1,562
Candidatus Marinimicrobia	0.00353	1,503
Thaumarchaeota	0.003472	1,478
Candidatus Hydrogenedentes	0.003051	1,299
Caldiserica	0.002873	1,223
Candidatus Aminicenantes	0.002598	1,106

Table S1. *Cont.*

Phylum	%	reads
Candidatus Poribacteria	0.002558	1,089
Dictyoglomi	0.002093	891
Candidatus Atribacteria	0.001823	776
candidate division Zixibacteria	0.001701	724
Candidatus Latescibacteria	0.001656	705
Elusimicrobia	0.00164	698
Candidatus Omnitrophica	0.00113	481
Candidatus Microgenomates	0.00105	447
Candidatus Calescamantes	0.000608	259
Candidatus Parcubacteria	0.000319	136
Candidatus Diapherotrites	0.000233	99
Candidatus Korarchaeota	0.000209	89
candidate division WWE3	0.000181	77
Nanoarchaeota	0.000101	43
Viruses	0.063997	27,245
cannot be assigned to a phylum	2.501812	1,065,087
unclassified	42.407274	18,053,886
	100.000001	
